# SARS-CoV-2–associated ssRNAs activate inflammation and immunity via TLR7/8

**DOI:** 10.1172/jci.insight.150542

**Published:** 2021-09-22

**Authors:** Valentina Salvi, Hoang Oanh Nguyen, Francesca Sozio, Tiziana Schioppa, Carolina Gaudenzi, Mattia Laffranchi, Patrizia Scapini, Mauro Passari, Ilaria Barbazza, Laura Tiberio, Nicola Tamassia, Cecilia Garlanda, Annalisa Del Prete, Marco A. Cassatella, Alberto Mantovani, Silvano Sozzani, Daniela Bosisio

**Affiliations:** 1 Department of Molecular and Translational Medicine, University of Brescia, Italy.; 2 IRCCS Humanitas Research Hospital, Rozzano (MI), Italy.; 3 Department of Medicine, Section of General Pathology, University of Verona, Italy.; 4 Department of Biomedical Sciences, Humanitas University, Pieve Emanuele (MI), Italy.; 5 The William Harvey Research Institute, Queen Mary University of London, London, United Kingdom.; 6 Laboratory Affiliated to Istituto Pasteur Italia-Fondazione Cenci Bolognetti, Department of Molecular Medicine, Sapienza University of Rome, Rome, Italy.; 7 IRCCS Neuromed, Pozzilli (IS), Italy.

**Keywords:** Immunology, Cytokines, Dendritic cells, Innate immunity

## Abstract

The inflammatory and IFN pathways of innate immunity play a key role in the resistance and pathogenesis of coronavirus disease 2019 (COVID-19). Innate sensors and SARS-CoV-2–associated molecular patterns (SAMPs) remain to be completely defined. Here, we identified single-stranded RNA (ssRNA) fragments from the SARS-CoV-2 genome as direct activators of endosomal TLR7/8 and MyD88 pathway. The same sequences induced human DC activation in terms of phenotype and function, such as IFN and cytokine production and Th1 polarization. A bioinformatic scan of the viral genome identified several hundreds of fragments potentially activating TLR7/8, suggesting that products of virus endosomal processing potently activate the IFN and inflammatory responses downstream of these receptors. In vivo, SAMPs induced MyD88-dependent lung inflammation characterized by accumulation of proinflammatory and cytotoxic mediators and immune cell infiltration, as well as splenic DC phenotypical maturation. These results identified TLR7/8 as a crucial cellular sensor of ssRNAs encoded by SARS-CoV-2 involved in host resistance and the disease pathogenesis of COVID-19.

## Graphical abstract

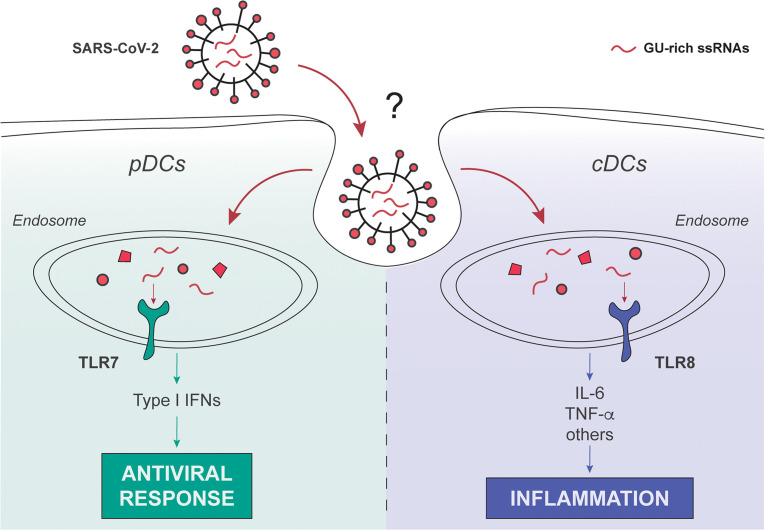

## Introduction

SARS-CoV-2 is a positive-sense single-stranded RNA (ssRNA) virus belonging to the family of Coronaviridae, also including the closely related Middle East respiratory syndrome coronavirus (MERS-CoV) and SARS-CoV (1). In a subgroup of patients, SARS-CoV-2 infection (coronavirus disease 2019, COVID-19) develops as acute respiratory distress syndrome featuring intense lung injury, sepsis-like manifestations, and multiorgan failure (2) associated with overt production of proinflammatory cytokines that directly correlates with poor prognosis (3). This clinical condition suggests that an overactive innate immune response may unleash virus-dependent immune pathology (4). Innate immune activation is also responsible for inducing the protective antiviral state, largely mediated by the release of type I IFNs. Indeed, inborn errors in type I IFN production and amplification (5) or preexisting blocking autoantibodies against members of the IFN family of cytokines (6) were found to correlate with unfavorable prognosis.

DCs act as crucial messengers linking innate and adaptive immunity against viral infections (7, 8). Within DC heterogeneity, plasmacytoid DCs (pDCs) play an important role as the major source of type I IFN in response to viral infection, while conventional DCs (cDCs) respond to a vast variety of pathogens by producing proinflammatory cytokines and are the main cells responsible for T cell activation (9–11). pDCs sense ssRNA viruses through TLR7 (12), an endosomal receptor activated by genomic fragments rich in guanine and uracil (GU rich), derived by endosomal processing of the virus independently of infection (13). By contrast, cDCs express the closely related TLR8 (14). Although TLR7 and TLR8 display high structural and functional homology and similar ligand specificity (15) and recruit the same signaling intracellular adaptor molecule, MyD88 (16), the signaling pathways of these 2 TLRs diverge in functional significance, with TLR7 more involved in the antiviral immune response and TLR8 mastering the production of proinflammatory cytokines. Both cDCs and pDCs were shown to be reduced in the blood of patients with severe acute COVID-19 (17, 18) as a possible result of cell activation (19), but the mechanisms of SARS-CoV-2 recognition and activation by innate immune cells still need to be identified. This study characterized SARS-CoV-2–associated molecular patterns (SAMPs) and identified the TLR7/8/MyD88 axis as a crucial pathway in the activation of human pDCs and cDCs.

## Results

### Identification of potential ssRNA SAMPs.

Based on previous work identifying RNA40, a GU-rich ssRNA from the U5 region of HIV-1, as a natural agonist of TLR7 and TLR8 (20) and on known features of TLR7/8 ligands (15, 21, 22), we searched for putative immunostimulatory sequences within the SARS-CoV-2 ssRNA genome. Our bioinformatic scan revealed 491 GU-rich sequences, among which more than 250 also bore at least 1 “UGUGU” IFN induction motif (IIM; refs. 15, 20, 21; Supplemental Table 1; supplemental material available online with this article; https://doi.org/10.1172/jci.insight.150542DS1).

We hypothesized that these sequences may represent as yet unidentified SAMPs responsible for viral recognition and immune activation via endosomal TLR triggering. The elevated number of sequences detected suggests that upon endosomal engulfment, the fragmentation of the SARS-CoV-2 genome may generate many TLR7/8-triggering sequences, thus displaying a high chance of contacting and activating the IFN and inflammatory responses downstream of these receptors.

To validate the stimulatory potential on innate immune cells, 2 representative sequences, SCV2-RNA1 and SCV2-RNA2, were chosen within the previous list, synthesized, and tested in in vitro and in vivo models of inflammation.

### ssRNA SAMPs activate human monocyte-derived DCs.

**Figure 1:**
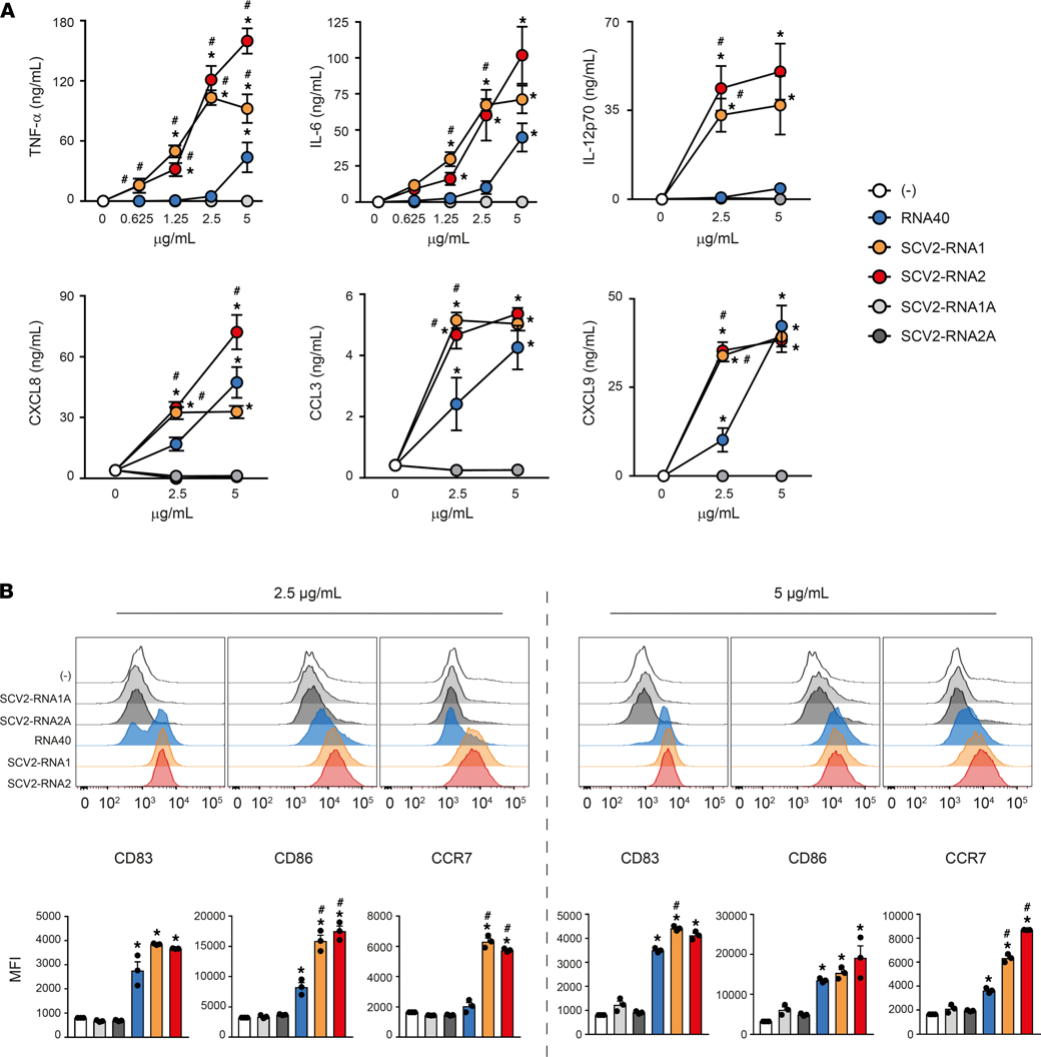
SAMPs activate cytokine secretion and phenotypical maturation of moDCs. (**A**) moDCs (2 × 10^6^/mL) were stimulated with increasing concentrations of the indicated viral RNAs or with vehicle alone (-) for 24 hours. The production of TNF-α, IL-6, IL-12p70, CXCL8, CCL3, and CXCL9 was evaluated by ELISA in cell-free supernatants. Data are expressed as mean ± SEM (*n =* 3). Results of SCV2-RNA1A and SCV2-RNA2A are superimposed in all graphs. (**B**) moDCs were stimulated as described in **A** and the surface expression of CD83, CD86, and CCR7 evaluated by FACS analysis. Data are expressed as representative cytofluorimetric profiles (upper panels) or as the mean ± SEM (*n =* 3) of the MFI (lower panels). (**A** and **B**) *P < 0.05 versus (-) by 1-way ANOVA with Dunnett’s post hoc test; ^#^*P <* 0.05 versus RNA40 by paired Student’s *t* test.

Monocyte-derived DCs (moDCs), a model of inflammatory cDCs expressing a wide variety of TLRs (7, 23–25), were treated with increasing concentrations of SCV2-RNA1 and SCV2-RNA2 along with HIV-1–derived RNA40 (20) used as a positive control. Uridine/adenosine–alternated (U/A-alternated) control sequences SCV2-RNA1A and SCV2-RNA2A were used as negative controls (see Methods). Figure 1A shows that both fragments efficiently activated cytokine secretion by moDCs. In particular, we observed potent induction of proinflammatory cytokines (TNF-α, IL-6), the Th1-polarizing cytokine IL-12 and chemokines recruiting polymorphonuclear neutrophils (CXCL8), myelomonocytic cells (CCL3), and Th1- and cytotoxic effector cells (CXCL9). Especially at low concentrations, SCV2-RNA1 and SCV2-RNA2 were more efficient than HIV-1–derived RNA40. In all experimental conditions, U/A-alternated SCV2-RNA1A and SCV2-RNA2A did not induce cytokine secretion. SCV2-RNA1 and SCV2-RNA2 also induced moDC phenotypical maturation in terms of CD83, CD86, and CCR7 expression (Figure 1B). Similar to cytokine secretion, upregulation of maturation markers by RNA40 was less effective. These results demonstrated that SCV2-RNA1 and SCV2-RNA2 behaved as SAMPs endowed with potent DC stimulatory capacity. Because of their similar potency, further experiments were carried out using a mixture of the 2 SAMPs (indicated as SCV2-RNA), a condition that may also better mimic physiological stimulation by multiple sequences derived from SARS-CoV-2 genome endosomal fragmentation.

### ssRNA SAMPs activate T cell responses.

**Figure 2:**
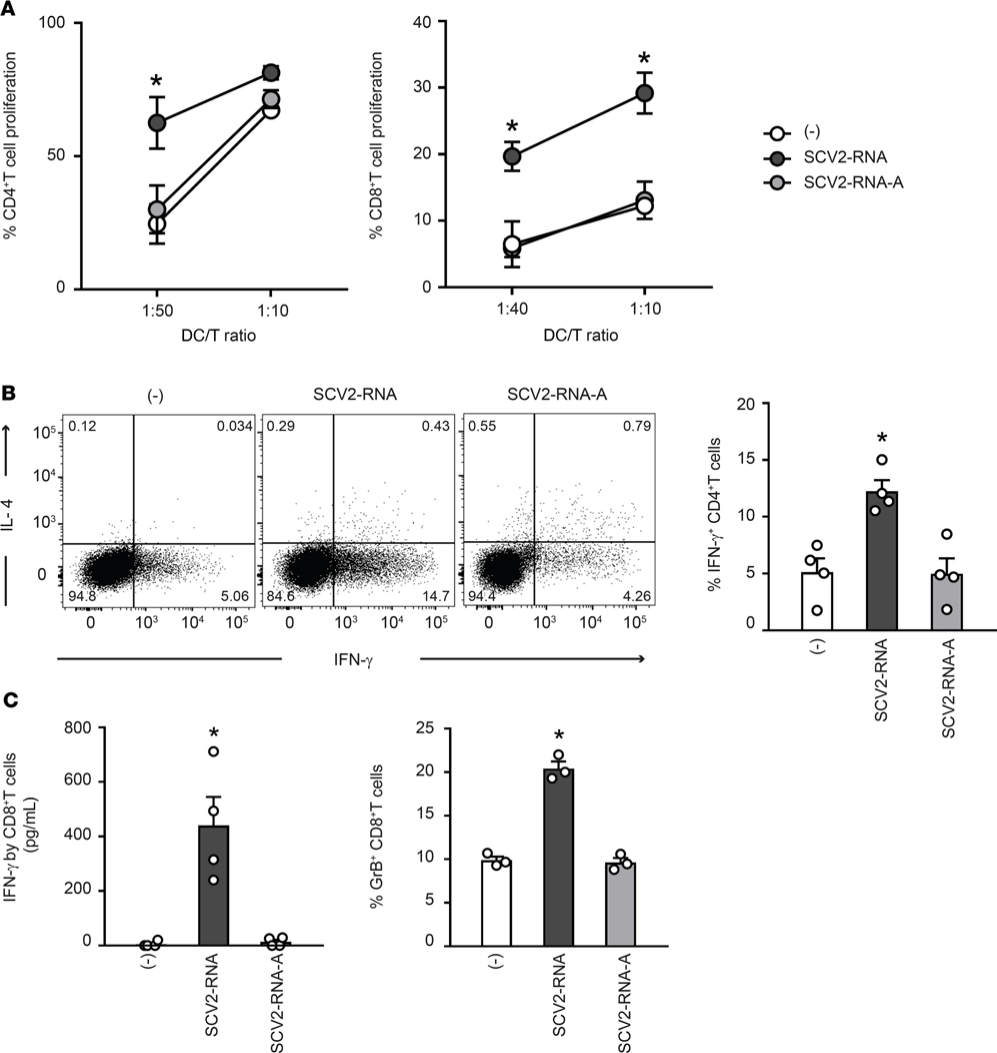
SAMP-activated DCs trigger T cell proliferation and functional activation. (**A**) moDCs were stimulated with vehicle (-) or with SCV2-RNA or the A-to-U–replaced SCV2-RNA-A (both at 5 μg/mL) for 24 hours. Activated moDCs were cocultured for 6 days with CFSE-stained allogenic naive CD4^+^ T cells or CD8^+^ T cells at the indicated DC/T cell ratio. Alloreactive T cell proliferation was assessed by measuring CellTrace-CFSE dye loss by flow cytometry. Data are expressed as mean ± SEM (*n =* 3) of the percentage of proliferating T cells. (**B**) moDCs stimulated as in **A** were cocultured for 6 days with allogenic naive CD4^+^ T cells (DC/T cell ratio 1:20). Intracellular IFN-γ and IL-4 were evaluated by FACS analysis. Left, dot plots from 1 representative experiment out of 4 are shown. Right, bar graphs from 4 independent experiments. Data are expressed as mean ± SEM of the percentage of IFN-γ–producing cells. (**C**) moDCs activated as in **A** were cocultured for 6 days with allogenic CD8^+^ T cells (DC/T cell ratio 1:10). IFN-γ production was evaluated by ELISA in cell-free supernatants and intracellular granzyme B (GrB) by FACS analysis. Data are expressed as mean ± SEM (*n =* 3). (**A**–**C**) *P < 0.05 versus (-) by 1-way ANOVA with Dunnett’s post hoc test.

The impact of SAMPs on the ability of DCs to stimulate T cell functions was investigated in coculture experiments of SAMP-activated DCs with allogeneic naive CD4^+^ and CD8^+^ T cells. Figure 2A shows that SAMP-activated DCs induced proliferation of naive CD4^+^ and CD8^+^ T cells. Activated CD4^+^ T cells produced IFN-γ but no IL-4, a typical Th1-effector phenotype (Figure 2B). Functional activation of CD8^+^ T cells was similarly demonstrated by the detection of secreted IFN-γ (Figure 2C) and the intracellular accumulation of granzyme B, a marker of a cytotoxic phenotype. None of these effects were observed when DCs were activated with U/A-alternated SAMPs.

These experiments demonstrated that phenotypical DC maturation induced by SAMPs (Figure 1B) was paralleled by the acquisition of T cell–activating capabilities. Thus, SAMPs have the ability to induce a Th1-oriented immune response.

### ssRNA SAMPs activate human primary DCs.

**Figure 3:**
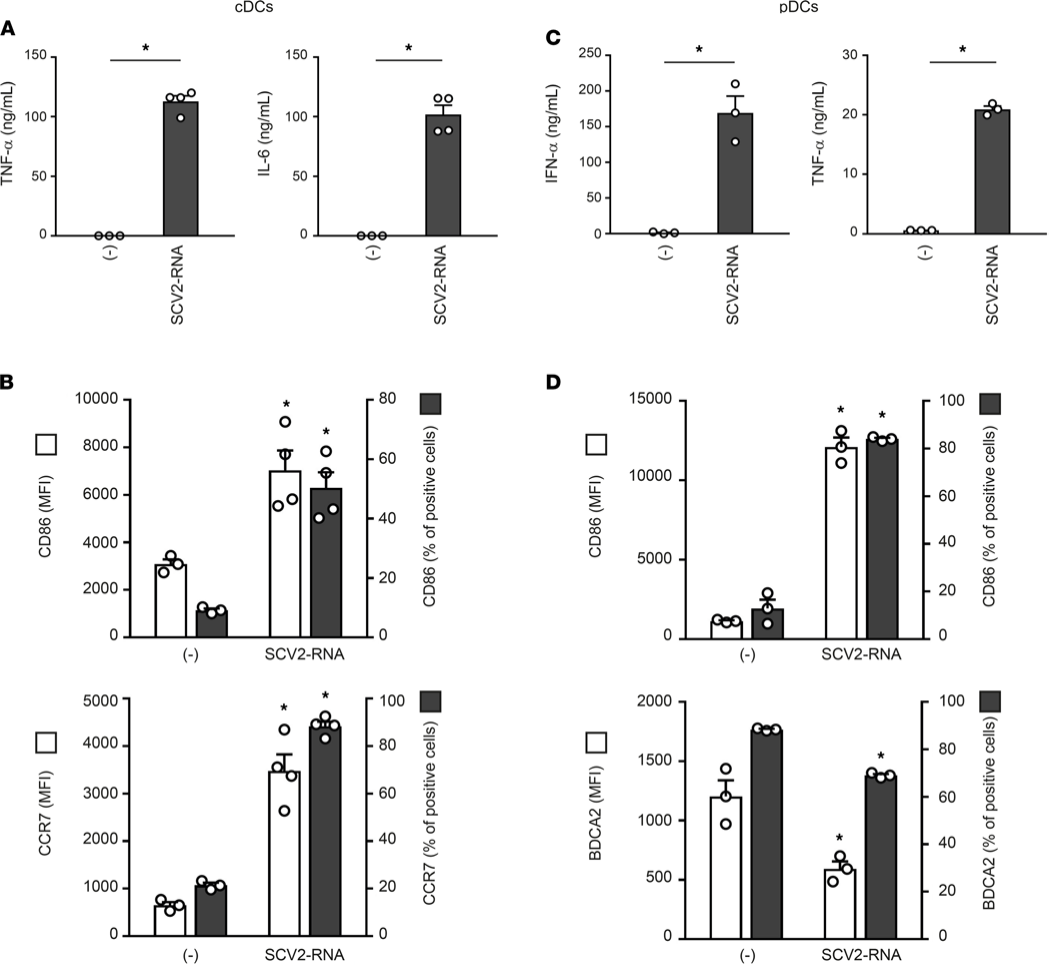
SAMPs activate cytokine secretion and phenotypical maturation in primary circulating DC subsets. cDCs (2 × 10^6^/mL) and pDCs (1 × 10^6^/mL) were stimulated with 5 μg/mL SCV2-RNA for 24 hours. (**A**–**C**) Cytokine secretion was evaluated by ELISA. Data are expressed as mean ± SEM (*n =* 3–4); *P < 0.05 versus (-) by paired Student’s *t* test. (**B**–**D**) Surface expression of CD86, CCR7, and BDCA2 was evaluated by FACS analysis. Data are expressed as mean ± SEM of the MFI (left *y* axis), as well as the mean ± SEM of the percentage of positive cells (right *y* axis) (*n =* 3–4); *P < 0.05 versus (-) by paired Student’s *t* test.

The ability of SCV2-RNAs to activate DCs was further investigated using primary circulating cDCs (comprising CD141^+^ cDC1 and CD1c^+^ cDC2) and BDCA2^+^ pDCs. SCV2-RNA efficiently induced the secretion of TNF-α and IL-6 (Figure 3A) and the expression of maturation markers, such as CD86 and CCR7 (Figure 3B), in cDCs. Similarly, SAMPs stimulated the release of IFN-α and TNF-α by pDCs (Figure 3C) as well as their maturation in terms of CD86 upregulation and BDCA2 reduction (Figure 3D). Similar to previous results, U/A-alternated control sequences did not activate cytokine production or maturation in either pDCs or cDCs (not shown).

### ssRNA SAMPs activate the TLR8/MyD88/NF-κB axis in moDCs.

**Figure 4:**
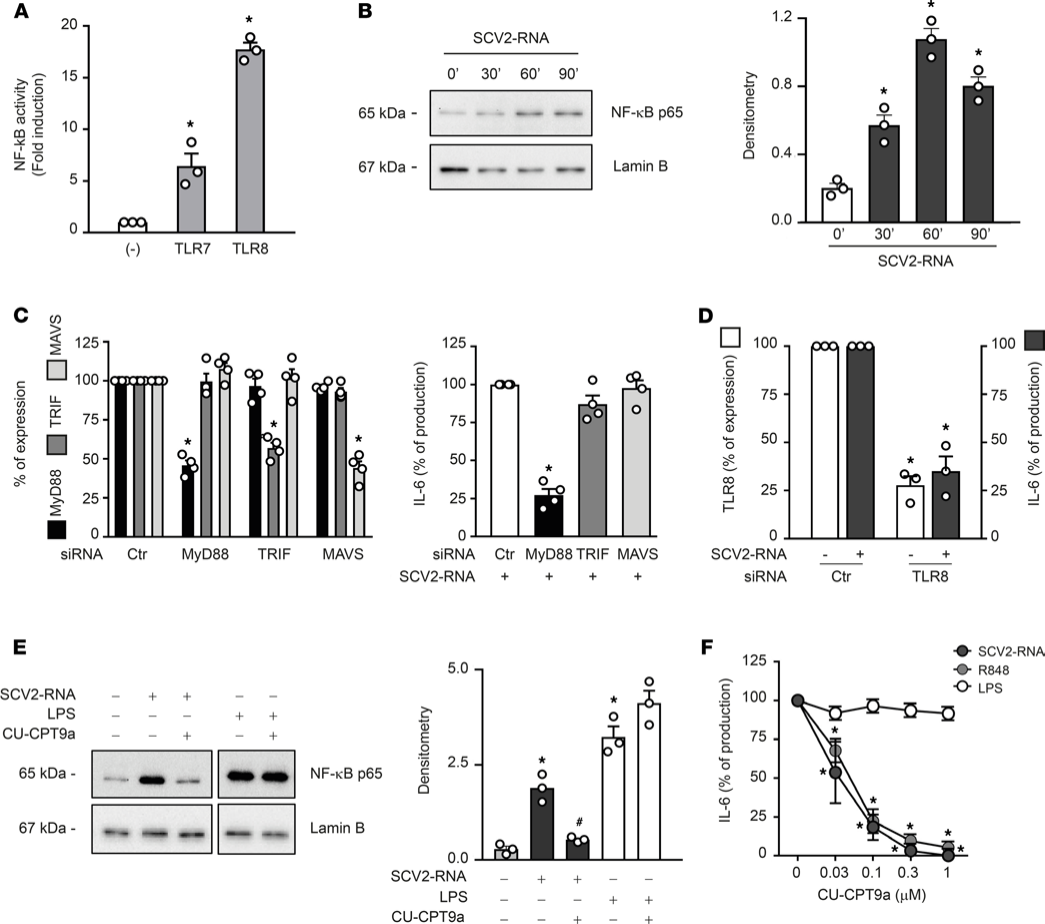
SAMPs activate the TLR8/MyD88/NF-κB axis in moDCs. (**A**) HEK293 cells stably transfected with human TLR7, TLR8, or luciferase alone (-) were stimulated with SCV2-RNA for 24 hours. NF-κB activation was evaluated as luciferase activity. Data are expressed as mean ± SEM (*n =* 3); *P < 0.05 versus (-) by 1-way ANOVA with Dunnett’s post hoc test. (**B**–**E**) moDCs were stimulated with SCV2-RNA as indicated (**B**) or pretreated with CU-CPT9a (1 μM) and then stimulated with SCV2-RNA or LPS for 1 hour (**E**). Nuclear extracts were blotted against NF-κB p65 and lamin B. One representative donor and densitometry of 3 donors are shown. *P < 0.05 versus untreated by 1-way ANOVA with Dunnett’s post hoc test; ^#^*P <* 0.05 versus “SCV2-RNA” by paired Student’s *t* test. (**C**, left panel) moDCs were transfected with indicated siRNAs and target gene expression evaluated by qPCR. Results depict percentage of target gene expression (mean ± SEM *n =* 4). (**C**, right panel) moDCs transfected with indicated siRNAs were stimulated with SCV2-RNA for 24 hours and IL-6 production evaluated by ELISA. Data are expressed as percentage of production (*n =* 4); *P < 0.05 versus “ctr siRNA” by 1-way ANOVA with Dunnett’s post hoc test. (**D**) moDCs were transfected with indicated siRNAs and the expression of TLR8 was evaluated by qPCR (left *y* axis, white bars). IL-6 production upon SCV2-RNA stimulation was evaluated by ELISA (right *y* axis, gray bars). Data (percentage of expression/production) represent the mean ± SEM (*n =* 3); *P < 0.05 versus respective “ctr” by paired Student’s *t* test. (**F**) moDCs were pretreated with CU-CPT9a, then stimulated as indicated for 24 hours and IL-6 production evaluated by ELISA. Data are expressed as percentage of production for each individual stimulation (*n =* 3); *P < 0.05 versus respective “0” by 1-way ANOVA with Dunnett’s post hoc test.

The cellular sensors responsible for SARS-CoV-2 detection by immune cells remain ill defined. To formally demonstrate the ability of SAMPs to functionally activate TLRs, experiments were performed in HEK293 cells stably transfected with human TLR7 and TLR8 together with an NF-κB reporter gene. Figure 4A depicts the SAMP-dependent activation of NF-κB and luciferase production in TLR7- and TLR8-expressing cells. NF-κB activation was also detected in SCV2-RNA–stimulated moDCs (Figure 4B). Since both TLR7 and TLR8 signal through the common adaptor MyD88, siRNA interference was performed in moDCs. Figure 4C shows that MyD88-specific siRNA could decrease by about 50% the levels of MyD88 mRNA, whereas the expression of the TLR3-related adaptor TRIF and RLR-related MAVS was not affected. Consistent with this result, IL-6 production by SCV2-RNA was also decreased, supporting a role for MyD88 in moDC activation by SCV2-RNA (Figure 4C). Because SAMPs, despite being designed to activate TLR7/8, may also engage other pattern recognition receptors (PRRs) expressed by moDCs, we also performed TRIF and MAVS siRNA interference. Although siRNAs efficiently and specifically inhibited the expression of target genes (Figure 4C), they failed to reduce IL-6 production by SCV2-RNA (Figure 4C). The predominant role of the MyD88/NF-κB pathway as compared with that of TRIF/MAVS/IRF-3 was also supported by the lack of SCV2-RNA–dependent induction of nuclear translocation of IRF-3, a transcription factor downstream of TLR3 and RLRs (not shown).

moDCs are known to respond mainly to TLR8 ligands and to express negligible levels of TLR7 mRNA (14, 23). mRNA and protein expression analysis of TLR7 and TLR8 confirmed selective expression of TLR8 in our experimental setting (Supplemental Figure 1, A and B). Based on this, we performed TLR8 siRNA in moDCs, showing a reduction in SCV2-RNA–dependent activation correlating to the levels of mRNA reduction (Figure 4D).

Next, moDCs were stimulated in the presence of CU-CPT9a, a specific TLR8 inhibitor (26). CU-CPT9a inhibited both NF-κB nuclear translocation (Figure 4E) and IL-6 production when cells were stimulated with SCV2-RNA or R848 (TLR7/8 ligand; Figure 4F). On the other hand, the TLR8 inhibitor did not affect the stimulation by LPS, a TLR4 ligand (Figure 4, E and F). Finally, we found that SCV2-RNA colocalized with TLR8 within moDCs (Supplemental Figure 1C).

### ssRNA SAMPs act as TLR7/8 ligands in primary DCs.

**Figure 5:**
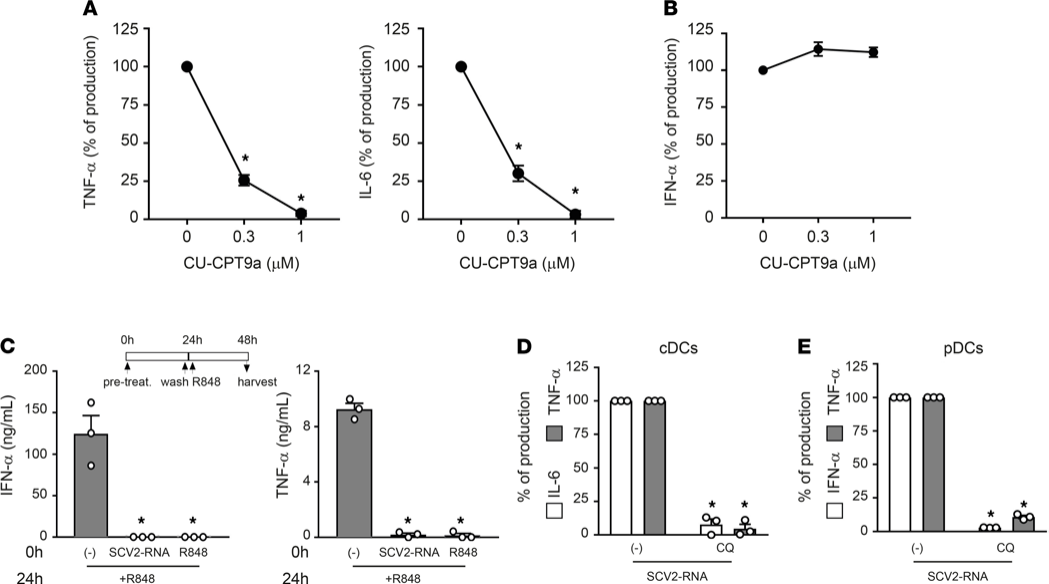
TLR7 and TLR8 are responsible for primary DC activation by SAMPs. cDCs (**A**) and pDCs (**B**) were pretreated with increasing concentration of CU-CPT9a and then stimulated with SCV2-RNA (5 μg/mL) for 24 hours. Secreted TNF-α, IL-6, and IFN-α were quantified by ELISA. Data are expressed as percentage of production (*n =* 3); *P < 0.05 versus “0” by 1-way ANOVA with Dunnett’s post hoc test. (**C**) pDCs were pretreated (0 hours) with SCV2-RNA (5 μg/mL) or R848 (1 μg/mL) or left untreated for 24 hours, washed, and restimulated with R848 for an additional 24 hours. Secreted IFN-α and TNF-α were quantified by ELISA. Data are expressed as mean ± SEM (*n =* 3); *P < 0.05 versus “(-)” by 1-way ANOVA with Dunnett’s post hoc test. (**D**) cDCs were pretreated for 1 hour with chloroquine (CQ, 10 μM) and then stimulated with SCV2-RNA (5 μg/mL) for 24 hours. Secreted IL-6 (white bars) and TNF-α (gray bars) were evaluated by ELISA. Data are expressed as percentage of production (*n =* 3); *P < 0.05 versus respective “(-) SCV2-RNA” by paired Student’s *t* test. (**E**) pDCs were pretreated for 1 hour with CQ (10 μM) and then stimulated with SCV2-RNA (5 μg/mL) for 24 hours. Secreted IFN-α (white bars) and TNF-α (gray bars) were quantified by ELISA. Data are expressed as percentage of production (*n =* 3); *P < 0.05 versus respective “(-) SCV2-RNA” by paired Student’s *t* test.

TLR7 and TLR8 display a mutually exclusive expression in primary DCs. Indeed, cDCs express TLR8 as their unique endosomal ssRNA receptor, whereas pDCs express TLR7 (14). Consistent with this, CU-CPT9a blocked the production of proinflammatory cytokines in cDCs (Figure 5A) but not in TLR7-expressing pDCs (Figure 5B). Our effort to block TLR7 signaling using commercially available receptor antagonists was unsuccessful since none of these inhibitors blocked TLR7 activation in pDCs stimulated with R848 or imiquimod (data not shown). As an alternative strategy to demonstrate the involvement of TLR7 in SCV2-RNA sensing, we performed TLR desensitization (21). pDCs were stimulated with SCV2-RNA or R848 or left untreated, washed, and then restimulated with R848. Figure 5C shows that upon restimulation, only untreated cells could respond to R848 in terms of IFN-α and TNF-α production as a result of TLR7 desensitization by its ligand R848 as well as by SCV2-RNA. The limited yield after blood DC purification hampered the use of siRNAs. However, the involvement of endosomal TLRs as SCV2-RNA receptors was further supported by the blocking of cytokine release in cDCs (Figure 5D) and pDCs (Figure 5E) by chloroquine, a drug known to block endosomal TLR triggering by interfering with endosomal acidification (27).

### ssRNA SAMPs induce DC activation and lung inflammation in vivo.

**Figure 6:**
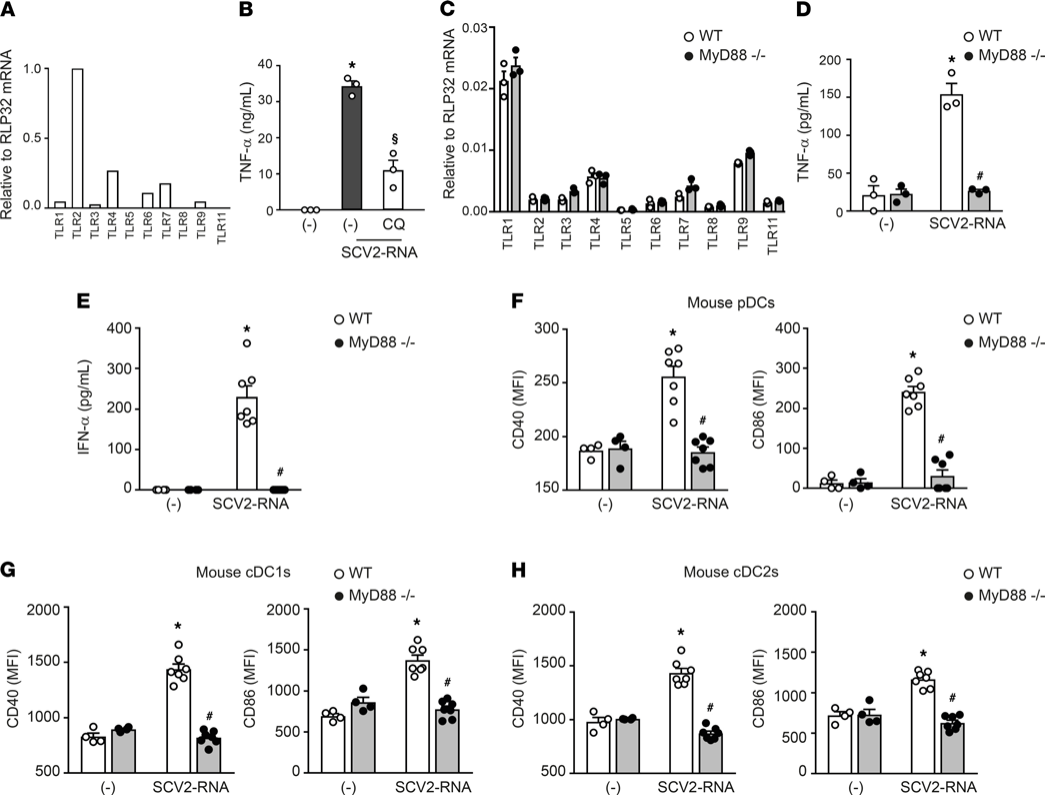
SAMPs activate murine cells in vitro and in vivo. (**A**) Expression of *TLR* mRNAs in RAW264.7 cells. Data are expressed as 2^–ΔCt^ relative to RPL32 of 1 representative experiment out of 3. (**B**) RAW264.7 (1 × 10^6^/mL) were pretreated for 1 hour with CQ (12.5 μM), then stimulated with 5 μg/mL SCV2-RNA or vehicle (-) for 24 hours. Secreted TNF-α was evaluated by ELISA. Data are expressed as mean ± SEM (*n =* 3); *P < 0.05 versus (-); ^§^P<0.05 versus “(-) SCV2-RNA” by paired Student’s *t* test. (**C**) Expression of TLR mRNAs in splenocytes from WT (white circle) or MyD88^–/–^ mice (black circle). Data are expressed as mean ± SEM (*n =* 3) of 2^–ΔCt^ relative to RPL32 of 1 representative experiment out of 3. (**D**) Splenocytes (3 × 10^6^/mL) from WT (white circle) or MyD88^–/–^ mice (black circle) were stimulated with 5 μg/mL SCV2-RNA or vehicle (-) for 24 hours. Secreted TNF-α was evaluated by ELISA. Data are expressed as mean ± SEM (*n =* 3); *P < 0.05 versus (-) or ^#^*P* < 0.05 versus “SCV2-RNA MyD88^–/–^” by paired Student’s *t* test . (**E**) Circulating IFN-α in WT (white circle) or MyD88^–/–^ mice (black circle) treated with SCV2-RNA or vehicle (-) for 6 hours. Data are expressed as mean ± SEM [(-) *n =* 4, SCV2-RNA *n =* 7]; *P < 0.05 versus (-) or ^#^*P* < 0.05 versus “SCV2-RNA MyD88^–/–^” by unpaired Student’s *t* test. of 1 representative experiment out of 3. (**F**–**H**) Activation of splenic pDCs (CD11c^int^MHC-II^+^B220^+^SiglecH^+^) (**F**), cDC1s (CD11c^+^MHC-II^+^CD8α^+^CD11b^–^) (**G**), or cDC2s (CD11c^+^MHC-II^+^CD8α^–^CD11b^+^) (**H**) from WT (white circle) or MyD88^–/–^ mice (black circle), treated with SCV2-RNA or vehicle (-) for 6 hours evaluated in terms of CD40 and CD86 expression. Data are expressed as mean ± SEM of the MFI [(-) *n =* 4, SCV2-RNA *n =* 7]; *P < 0.05 versus (-) or ^#^*P* < 0.05 versus “SCV2-RNA MyD88^–/–^” by unpaired Student’s *t* test.

To address the capacity of SAMPs to induce inflammation and immune activation in vivo, we first investigated whether SAMPs can also trigger murine TLR7, the only GU-rich ssRNA-sensing TLR in mice (20). Murine TLR7 activation by SAMPs could be hypothesized based on previous studies demonstrating activation of human TLR7/8 and murine TLR7 by common GU-rich ssRNA ligands (20, 28). In support of this, we showed that TLR7-expressing RAW264.7 cells (Figure 6A) responded to SAMP stimulation by producing TNF-α, an effect that was reduced by chloroquine pretreatment (Figure 6B), confirming that SCV2-RNA activated murine cells, presumably via TLR7. In addition, splenocytes from MyD88^–/–^ mice did not respond to SCV2-RNA stimulation either in terms of proinflammatory cytokine production (Figure 6D) or TLR modulation (Supplemental Figure 2), despite expressing similar levels of TLRs as compared with WT mice (Figure 6C).

**Figure 7:**
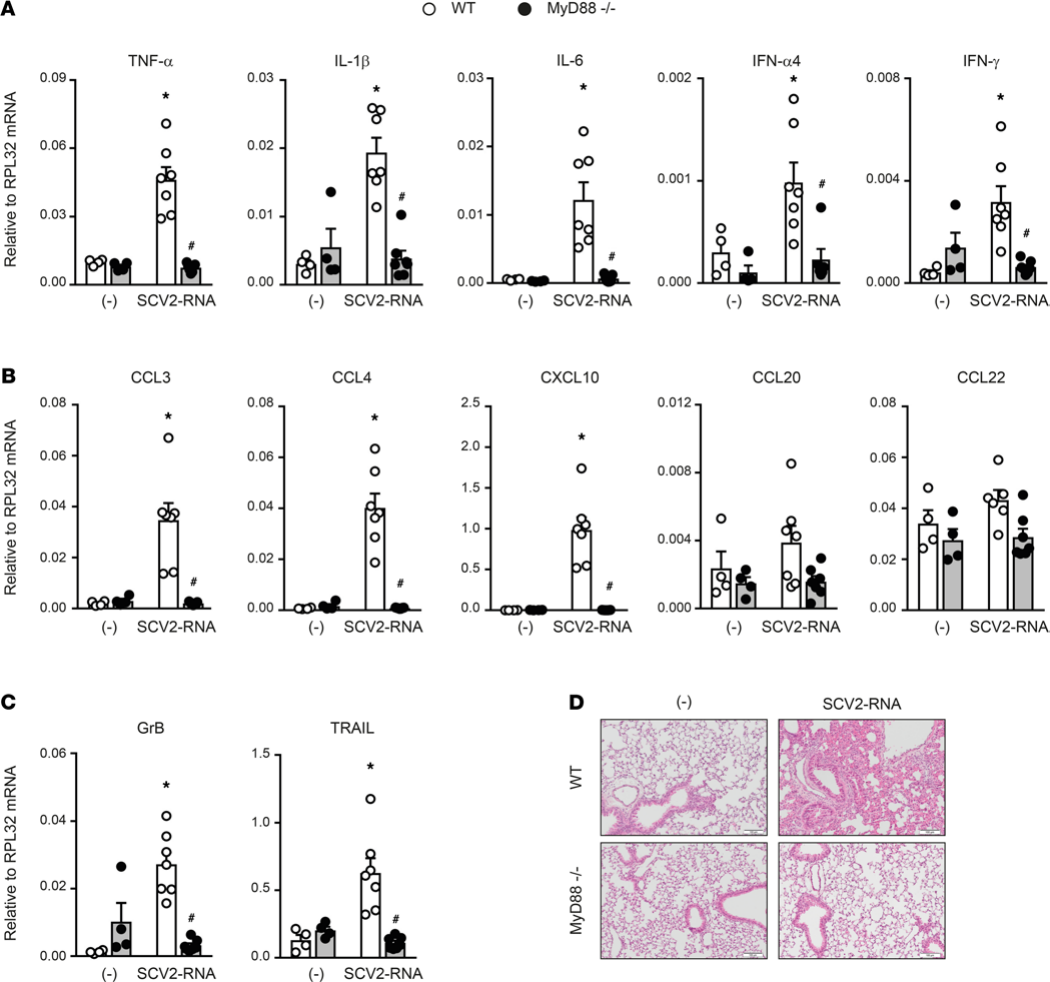
SAMPs induce inflammation in vivo. (**A**–**C**) Real-time PCR for cytokines, chemokines, and effector proteins in lungs of WT (white circle) or MyD88^–/–^ (black circle) treated or not with SCV2-RNA for 6 hours. Data are expressed as mean ± SEM [(-) *n =* 4, SCV2-RNA *n =* 7] of 2^–ΔCt^ relative to housekeeping mRNA (*RPL32*); *P < 0.05 versus (-) or ^#^*P* < 0.05 versus “SCV2-RNA MyD88^–/–^” by unpaired Student’s *t* test of 1 representative experiment out of 3. (**D**) Histological evaluation of lungs from WT or MyD88^–/–^ mice treated or not with SCV2-RNA for 6 hours. Image shows 1 section out of the 3 longitudinal serial sections performed of 1 representative left lung out of 7. Scale bars: 100 μm.

Based on these results, C57Bl6/J WT and MyD88^–/–^ mice were i.v. injected with SAMPs or vehicle and euthanized 6 hours later. A significant increase of type I IFN was detected in the sera of WT SAMP-treated mice, indicating systemic immune activation (Figure 6E). Consistent with this, SAMPs induced the upregulation of CD40 and CD86 on splenic pDCs (CD11c^int^MHC-II^+^B220^+^SiglecH^+^; Figure 6F). Activation of splenic cDC1s (CD11c^+^MHC-II^+^ CD8α^+^CD11b^–^) and cDC2s (CD11c^+^MHC-II^+^ CD8α^–^CD11b^+^) was also detected (Figure 6, G and H). Figure 7A shows that SAMP treatment induced the expression of proinflammatory cytokines TNF-α, IL-1β, and IL-6 and of IFN-α and IFN-γ in the lung. In addition, a marked increase in the expression of chemokines active on myeloid and Th1-effector cells (i.e., CCL3, CCL4, and CXCL10) was also detected. Conversely, CCL20 and CCL22, 2 chemokines active in Th17 and Th2 T cell recruitment, were not increased (Figure 7B). We could also detect the accumulation of molecules involved in cytotoxic tissue damage such as granzyme B and TRAIL (Figure 7C) that, given the short kinetics of stimulation, may reflect the recruitment of NK cells to the lungs. The increase in CD45 and MHC-II levels (data not shown) further suggested immune cell infiltration, which was confirmed by histological analysis. Lung histology revealed a marked infiltration of inflammatory cells into peribronchial and perivascular connective tissue and alveolar septal thickening in SAMP-treated mice (Figure 7D). On the contrary, SAMP administration to MyD88^–/–^ mice did not induce any inflammatory response, including the increase of circulating levels of type I IFN, DC maturation, and the generation of a lung infiltrate (Figure 6, D–H, and Figure 7). These data extend to the in vivo condition the observation that SAMPs use a TLR/MyD88-dependent pathway to trigger a type I IFN/proinflammatory activation program and highlight the lung as a primary target organ.

## Discussion

Here, we report that 2 short sequences within the ssRNA genome of SARS-CoV-2 activate the production of type I IFNs and the T cell–activating ability of human DCs by triggering endosomal TLR7 and TLR8. Of note, these sequences represent prototypical examples of the several hundreds of potential TLR ligands identified by SARS-CoV-2 genome scan. This finding is in line with previous work demonstrating a 20-fold higher density of GU-rich fragments in the closely related SARS-CoV as compared with HIV-1 (29) and with a recent bioinformatic study showing that SARS-CoV-2 encodes a number of such fragments even larger than SARS-CoV (30). Thus, endosomal processing of SARS-CoV-2 nucleic acids may give rise to multiple fragments endowed with the property to trigger innate immune activation.

TLR7/8 are sensors of ssRNA viruses including coronaviruses. In the past, TLR7-dependent recognition of MERS-CoV and human and murine pDC activation was demonstrated (31). In addition, murine coronavirus activated protective type I IFN production by TLR7-expressing murine pDCs (32), and the ssRNA SARS-CoV genome was shown to induce TLR7/8-dependent cytokine secretion by human PBMCs and RAW264.7 murine cells (29). By contrast, the involvement of TLR7/8 in the immune response against SARS-CoV-2 and role in COVID-19 pathogenesis and therapeutic potential has only been hypothesized (33). Notably, very rare loss-of-function variants of TLR7 in 2 independent families were associated with severe COVID-19 in males (34). Thus, our report on the ability of SAMPs to activate the TLR7/8 and MyD88 pathways provides the missing link between clinical evidence and molecular knowledge on the cellular sensors for SARS-CoV-2 detection. Viral recognition by endosomal TLRs takes place before and independently of infection as a consequence of pathogen endocytosis (13). Indeed, pDCs were reported to be resistant to infection, although they were activated by SARS-CoV-2 (35). This is an important process that gives innate immune cells the opportunity to activate early antiviral response. One limitation of our experimental approach is that it does not shed light on the actual triggering of endosomal TLRs during active SARS-CoV-2 infection. However, this is a likely event based on the reported SARS-CoV-2–dependent pDC activation, which uses TLR7 as the main ssRNA receptor (12, 35). In addition, endosomal TLRs expressed by innate immune cells were shown to be activated by viral RNAs packaged within extracellular vesicles by infected tissue cells (36), a mechanism that is mimicked by SCV2-RNA delivery by liposomal particles. Indeed, in another experimental setting, liposome-delivered ssRNA40 from HIV-1 activated human macrophages via TLR8 in a way that recapitulated intact HIV-1 administration (37). It remains to be elucidated whether SARS-CoV-2 uptake for endosomal processing is a direct process or mediated by receptors, such as ACE2 or CD147 (38).

DCs are heterogeneous cells that master activation of inflammation and antiviral responses, adaptive immune responses, and tolerance (7, 8). These functions are largely shared among different phenotypical and functional DC subsets (39). Indeed, pDCs are the major producers of type I IFNs in response to viral infections (10–12), while cDCs, and cDC2s in particular, sustain inflammation via cytokine secretion and activate naive T cells (39). Notably, this specialization mirrors the respective expression and function of TLR7 and TLR8 (14). The protective role of TLR7 and type I IFNs in life-threatening COVID-19 has been documented based on the clinical outcome of patients with inborn errors in type I IFN immunity, producing blocking autoantibodies against different types of type I IFNs (5, 6) or expressing loss-of-function variants of TLR7 (34). Therefore, SAMPs may represent one of the essential signals in the activation of an IFN response and Th1-oriented adaptive immunity (40, 41). In this regard, it is of note that SARS-CoV-2 infection affected the number of pDCs in vivo (17, 18) and primary virus isolates induced the activation of pDCs in vitro (35). By contrast, an aggravated inflammatory response causes damage to the host and frequently advances to acute respiratory distress syndrome in patients with severe COVID-19. Here, we showed that the activation program induced by SAMPs was not restricted to type I IFNs but encompassed the production of proinflammatory cytokines and the generation of Th1-oriented responses, which may contribute to the exuberant proinflammatory response observed in life-threatening COVID-19 (42). Whether TLR8 or cDC overactivation or genetic variants are involved in this process is difficult to speculate, and more studies on selected patient cohorts are needed. However, TLR7 and TLR8 selective agonists or antagonists, inducing antiviral IFN response and/or controlling inflammation, deserve consideration and have entered phase II clinical trials as interesting therapeutic options to control the different manifestations of COVID-19 (ClinicalTrials.gov NCT04448756). ssRNA-sensing TLRs are expressed by cells other than DCs, such as macrophages, and by peripheral tissues, such as the lung, bronchus, rectum, and cerebral cortex (38). Thus, other cells may contribute to the complex balance of protective versus detrimental immune activation (4). Finally, since the magnitude of TLR activation differs in individuals, such as elderly people, differences in TLR activation may help explain differences in the quality of the antiviral immune response independently of SAMP potency (39).

By all means, other SAMPs and damage-associated molecular patterns (DAMPs) as well as the simultaneous engagement of different PRRs are likely to contribute to COVID-19–associated protective response and cytokine storm, including cytosolic sensors, such as retinoid-inducible gene-I–like receptors (43), IFN-induced proteins with tetratricopeptide repeats, or members of a large group of RNA-binding molecules with poorly defined ligand specificity (43). A search for specific candidate ligands of cytosolic RNA sensors was hampered because of the scarce definition of their ligand consensus sequences. However, the finding that SARS-CoV-2 can evade innate immune restriction provided by intracellular RNA sensors via methylation of the 5′ end of its cellular mRNAs (44) further reinforces the role for TLRs as crucial sentinels and regulators of immune response to SARS-CoV-2 infection. SARS-CoV-2 is known to induce inflammasome assembly, although the exact mechanism still needs to be characterized (45, 46). Since intracellular nucleic acid sensors are known to activate inflammasomes (47), and TLR activation is intimately connected with inflammasome functions (48, 49), it is possible that SCV2-RNAs used in this study may also contribute to activation of this pathway.

In conclusion, this work has described SARS-CoV-2 as a potential powerful source of immunostimulatory nucleic acid fragments and has identified SARS-CoV-2–specific pathogen-associated molecular patterns (PAMPs) endowed with the ability to promote inflammation and immunity triggering TLR7 and TLR8. Based on previous studies demonstrating a) the crucial protective role of type I IFNs against COVID-19 (5, 6), b) the crucial protective role of TLR7 against life-threatening SARS-CoV-2 infection (34), and c) pDC activation in vitro by SARS-CoV-2 (35), we believe that our findings fill a gap in the understanding of SARS-CoV-2 host-pathogen interaction.

## Methods

### Identification of potential TLR7/8-triggering ssRNA PAMPs.

The reference SARS-CoV-2 genome (NC_045512, positive strand) was scanned for GU-rich ssRNA fragments with the SequenceSearcher tool in the Fuzzy mode (50). We defined “GU-enriched sequences” as short strings with a maximal length of 20 bp that were composed of GU and/or UG pairs for more than 40% of their length. The identified 491 GU-rich sequences were further selected based on the content of at least 1 UGUGU IIM (ref. 21; see Supplemental Table 1). Within this list, the following were selected based on the particular enrichment in IIM (21) and synthesized by Integrated DNA Technologies (IDT) for subsequent studies: SCV2-RNA1 5′-UGCUGUUGUGUGUU*U-3′ (genome position: 15692-15706); SCV2-RNA2 5′-GUGUGUGUGUUCUGUUAUU*G-3′ (genome position: 20456-20475; *indicates a phosphorothioate linkage). These sequences were checked for uniqueness with BLAST (https://blast.ncbi.nlm.nih.gov/Blast.cgi?PROGRAM=blastn&PAGE_TYPE=BlastSearch&LINK_LOC=blasthome) selecting RNA viruses (taxid: 2559587) as organism in the “search set” window”. Two additional sequences were synthesized in which U was substituted with A in order to impair TRL7/8 stimulation (SCV2-RNA1A and SCV2-RNA2A; refs. 15, 20).

### Cell preparation and culture.

PBMCs were obtained by density gradient centrifugation and monocytes were subsequently purified by immunomagnetic separation using anti-CD14–conjugated magnetic microbeads (Miltenyi Biotec) according to the manufacturer’s protocol and as previously published (23). Briefly, monocytes were cultured for 6 days in tissue culture plates in complete medium (RPMI 1640 supplemented with 10% heat-inactivated, endotoxin-free FBS, 2 mM L-glutamine, penicillin, and streptomycin; all from Gibco, Thermo Fisher Scientific) in the presence of 50 ng/mL GM-CSF and 20 ng/mL IL-4 (Miltenyi Biotec). Untouched peripheral blood cDC1 and cDC2 (cDCs) and pDCs were obtained from PBMCs after negative immunomagnetic separation with the Myeloid Dendritic Cell Isolation kit (Miltenyi Biotec) and the Plasmacytoid Dendritic Cell Isolation kit II (Miltenyi Biotec), respectively. pDCs were cultured in completed RPMI medium with 20 ng/mL IL-3 (Miltenyi Biotec). RAW264.7 cells were purchased from American Type Culture Collection (ATCC) and cultured in DMEM complemented with 10% FBS.

### Cell stimulation.

Complexation of RNA with DOTAP Liposomal Transfection Reagent (Roche) was performed as previously described (21). Briefly, 5 μg RNA (either SCV2-RNA1 alone, SCV2-RNA2 alone, or 2.5 μg SCV2-RNA1 + 2.5 μg SCV2-RNA2 to obtain SCV2-RNA) in 50 μL HBS buffer (20 mM HEPES, 150 mM NaCl, pH 7.4) was combined with 100 μL DOTAP solution (30 μL DOTAP plus 70 μL HBS buffer) and incubated for 15 minutes at room temperature. Where indicated, cells were pretreated for 1 hour with chloroquine or CU-CPT9a or stimulated with the following TLR agonists: LPS (100 ng/mL) and R848 (1 μg/mL) (all from InvivoGen).

### siRNA silencing in moDCs.

Differentiating monocytes at day 2 of culture were transfected with MyD88 or TRIF or MAVS or TLR8 Silencer Select Validated siRNA or with a control siRNA (all at 50 nM final concentration; Ambion, Thermo Fisher Scientific) using Opti-MEM I reduced serum medium and Lipofectamine RNAiMAX transfection reagent (Thermo Fisher Scientific) as previously described (51). Transfected cells were incubated for 72 hours and then stimulated for 24 hours with SCV2-RNA. The effects of mRNA silencing by siRNA were investigated by quantitative PCR (qPCR) using specific QuantiTect primer assay (Qiagen).

### Cytokine detection.

TNF-α, IL-6, IL-12p70, CXCL8, CXCL9, CCL3, and mouse TNF-α were measured by ELISA (R&D Systems). Human IFN-α was detected using specific Module Set ELISA kit (eBioscience). Mouse IFN-α was measured by a bioluminescence kit (InvivoGen). All assays were performed on cell-free supernatants according to the manufacturer’s protocol.

### Flow cytometry.

Human and mouse DCs were stained with the following antibodies from Miltenyi Biotec or as specified: Vioblue-conjugated anti–human CD86 (clone FM95), PE-conjugated anti–human CD83 (clone REA714), FITC-conjugated anti–human BDCA2 (clone AC144), APC-conjugated anti–human CCR7 (clone REA546), VioGreen-conjugated anti–mouse CD45 (clone REA737), VioBlue or FITC-conjugated anti–mouse MHC-II (clone REA564), PerCP-Vio 700–conjugated anti–mouse CD11c (clone REA754), PE-conjugated anti–mouse SiglecH (clone 551.3D3), PE-Vio 615–conjugated anti–mouse CD11b (clone REA592), VioBlue-conjugated anti–mouse CD8a (cloneREA601), PE-Vio 770–conjugated anti–mouse B220 (clone RA3-6B2), APC-conjugated anti–mouse CD3 (clone REA641), APC-conjugated anti–mouse CD19 (clone REA749), APC-conjugated anti–mouse CD49b (clone DX5), APC-conjugated anti–mouse Ly6G (clone REA526), PE-conjugated anti–mouse CD40 (clone REA965), FITC-conjugated anti–mouse CD40 (clone HM40-3, BioLegend), and APC-CY7–conjugated anti–mouse CD86 (clone GL-1, BioLegend). Samples were read on a MACSQuant Analyzer (Miltenyi Biotec) and analyzed with FlowJo (Tree Star Inc.). For intracellular detection of granzyme B, cells were fixed and permeabilized using the Inside Stain kit (Miltenyi Biotec) and stained with APC-conjugated anti–granzyme B (clone REA226, Miltenyi Biotec). Cell viability was assessed by LIVE/DEAD staining according to the manufacturers’ instructions (Molecular Probes, Thermo Fisher Scientific). The gating strategy of mouse DCs was as follows: cells were first defined from FSC-A/SSC-A over doublet exclusion and gating on live CD45^+^ LIN^−^ cells (defined as CD3/CD19/CD49b/Ly6G^–^). Therefore, pDCs were identified as CD11c^int^MHC-II^+^B220^+^SiglecH^+^ cells; cDC1s as CD11c^+^MHC-II^+^CD8α^+^CD11b; cDC2s as CD11c^+^ MHC-II^+^ CD8α^–^CD11b^+^ (52).

### NF-κB luciferase reporter assay.

TLR-specific activation assays were performed using human HEK293 cells (ATCC) expressing luciferase under control of the NF-κB promoter and stably transfected with human TLR7 and TLR8 as previously described (21). Briefly, 25,000 cells were seeded in complete DMEM without antibiotics in 96-well plates for 24 hours and then stimulated with 10 μg/mL SCV2-RNA for an additional 24 hours. After stimulation, cells were lysed using ONE-Glo EX Luciferase Assay System (Promega) according to the manufacturer’s recommendations and assayed for luciferase activity using the EnSightMultimode Plate Reader (PerkinElmer). HEK293 cells were maintained in DMEM supplemented with 10% FBS and specific selection antibiotics were added.

### SDS-PAGE and Western blot.

Following the indicated stimulations, moDCs were washed twice with PBS and lysed in L1 buffer (50 mM Tris-HCl, pH 8.0; 2 mM EDTA; 0.1% NP-40; 10% glycerol) supplemented with inhibitors (1 mM Na_3_OV_4_, 2 mM DTT, 1 mM NaF, 1 mM PMSF, and protease inhibitor cocktail; all from MilliporeSigma) to separate cytoplasmic proteins. Nuclear pellets were washed twice with L1 buffer with inhibitors and then lysed in NP-40 lysis buffer (50 mM Tris-HCl, pH 8.0; 250 mM NaCl; 1 mM EDTA; 0.1% NP-40; 10% glycerol) with inhibitors. For the analysis of TLR expression, moDCs and HEK293-transfected cells were lysed in NP-40/Triton X-100 lysis buffer (10 mM Tris-HCl, pH 7.9; 150 mM NaCl; 0.6% NP-40; and 0.5% Triton X-100) supplemented with inhibitors. Equal amounts of extracts were analyzed through SDS-PAGE followed by Western blotting with antibodies against NF-κB p65 (rabbit polyclonal, C-20, sc-372, Santa Cruz Biotechnology), lamin B (goat polyclonal, C-20, 6216, Santa Cruz Biotechnology), TLR7 (rabbit monoclonal, 5632, Cell Signaling Technology), TLR8 (rabbit monoclonal, 11886, Cell Signaling Technology), and β-actin (mouse monoclonal, C4, sc-47778, Santa Cruz Biotechnology). Protein bands were detected with SuperSignal West Pico Chemiluminescent Substrate (Pierce) and quantified by computerized image analysis using Image Lab software (Bio-Rad). Data were normalized based on β-actin or lamin B content.

### Immunofluorescence.

moDCs were incubated with Atto-488–tagged SCV2-RNA1 (synthesized by Bio-Fab research) for 15 minutes, fixed with 4% paraformaldehyde (Pierce) for 10 minutes, and then seeded on glass slides by cytospin. After permeabilization with 100% cold methanol for 5 minutes, cells were labeled with a rabbit monoclonal anti–human TLR8 (11886, Cell Signaling Technology). A conjugate Alexa Fluor 594 anti-rabbit (A-11072, Thermo Fisher Scientific) was used as a secondary antibody. Glass slides were mounted using Prolong antifade with DAPI (Thermo Fisher Scientific). Cells were analyzed under a Zeiss Observer Z1 epifluorescence microscope equipped with a Plan-Apochromat 100×/1.4 numerical aperture oil objective and ApoTome2 imaging system for optical sectioning. *Z*-stack images were elaborated through AxioVision 3D and extended focus modules.

### T cell proliferation assay.

Experiments using T cells were conducted according to the “Minimal Information about T Cell Assays” (MIATA) guidelines. Allogenic naive CD4^+^ T cells and CD8^+^ T cells were isolated from buffy coats using the naive CD4^+^ T cell isolation kit II (Miltenyi Biotec) and CD8^+^ T cell isolation kit (Miltenyi Biotec), respectively. Purified T cells were counted by flow cytometry and labeled with CellTrace-CFSE (Molecular Probes, Thermo Fisher Scientific) at a final concentration of 5 μM. Subsequently, T cells (1 × 105 cells/well) were cocultured with graded numbers of allogeneic moDCs in 96-well round-bottom culture plates in complete RPMI medium. After 6 days, alloreactive T cell proliferation was assessed by measuring the loss of the dye CellTrace-CFSE upon cell division using flow cytometry. Positive controls of T cell proliferation were routinely performed using IL-2 plus phytohemagglutinin (PHA). Response definition criteria were defined post hoc. Dead cells were excluded by LIVE/DEAD staining according to the manufacturer’s instructions. These experiments were performed using general research investigative assays. Raw data can be provided upon request.

### Analysis of T cell cytokine production.

After 6 days of coculture, helper T cells were restimulated with 200 nM PMA (Sigma-Aldrich) plus 1 μg/mL of ionomycin (Sigma-Aldrich) for 5 hours. Brefeldin A (5 μg/mL, Sigma-Aldrich) was added during the last 2 hours. For intracellular cytokine production, cells were fixed and permeabilized with Inside Stain kit (Miltenyi Biotec) and stained with FITC-conjugated anti–IFN-γ (clone 45-15, Miltenyi Biotec) and PE-conjugated anti–IL-4 (clone 7A3-3, Miltenyi Biotec) following the manufacturer’s recommendations. For CD8^+^ T cells, after 6 days of coculture, IFN-γ production was assessed in the culture supernatants by ELISA (R&D Systems). Response definition criteria were defined post hoc. These experiments were performed using general research investigative assays. Raw data can be provided upon request.

### In vivo experiments.

MyD88^–/–^ mice (C57Bl6/J background) were provided by S. Akira (Laboratory of Host Defense, Immunology Frontier Research Center, Osaka University). WT C57Bl6/J mice were purchased from Charles River Laboratories. All mice were housed in the specific pathogen–free animal facility of the Department of Medicine, University of Verona. Sex- and age-matched WT and MyD88^–/–^ mice (8–10 weeks old) were anesthetized with isoflurane and i.v. injected in the retro-orbital vein with 300 μL DOTAP/SCV2-RNA mixture (20 μg/mouse) or with DOTAP alone. After 6 hours, mice were euthanized and lungs, spleen, and blood were harvested. Briefly, lungs were collected upon intracardiac perfusion with cold PBS. Left lung lobes were formalin-fixed for 24 hours, dehydrated, and paraffin-embedded for histological analysis. Right lungs were immediately frozen at –80°C and used for real-time PCR. Spleens were mechanically and enzymatically treated to obtain a single-cell suspension for cytofluorimetric and real-time PCR analysis.

### Lung histological analysis.

Histology was performed on 3 longitudinal serial sections (150 μm apart, 4 μm in thickness) from each left lung, stained with H&E, and scanned by VS120 Dot Slide BX61 virtual slide microscope (Olympus Optical) as previously described (53).

### qPCR.

RNA was extracted using TRIzol reagent and treated with DNAse according to the manufacturer’s instructions, and reverse transcription was performed using random hexamers and Moloney Murine Leukemia Virus Reverse Transcriptase (MMLV RT) (all from Thermo Fisher Scientific). The SsoAdvanced Universal SYBR Green Supermix (Bio-Rad) was used according to the manufacturer’s instructions. Reactions were run in triplicate on a StepOne Plus Real-Time PCR System (Applied Biosystems) and analyzed by StepOne Plus Software (Version 2.3, Applied Biosystems). Sequences of gene-specific primers are listed in Supplemental Table 2. Gene expression was normalized based on mouse RPL32 or human hypoxanthine phosphoribosyltransferase (HPRT) mRNA content.

### Statistics.

Sample group normality was confirmed by Shapiro-Wilk test before application of parametric statistical analysis. Statistical significance among the experimental groups was determined using 2-tailed paired or unpaired Student’s *t* test or 1-way ANOVA with Dunnett’s post hoc test (GraphPad Prism 7) as indicated in each figure legend. *P* less than 0.05 was considered significant; *n* indicates the number of biological replicates and is specified in each figure legend.

### Study approval.

Buffy coats from blood donations of anonymous healthy donors were obtained and preserved by the Centro Trasfusionale, Spedali Civili of Brescia according to the Italian law concerning blood component preparation and analysis. Procedures involving animal handling and care conformed to protocols approved by the University of Verona in compliance with national (D.L. N.116, G.U., Supplemental 40, 18-2-1992 and N. 26, G.U. March 4, 2014) and international law and policies (EEC Council Directive 2010/63/EU, OJ L 276/33, 22-09-2010; NIH Guide for the Care and Use of Laboratory Animals, National Academies Press, 2011). The study was approved by the Italian Ministry of Health (approval 339/2015-PR). All efforts were made to minimize the number of animals used and their suffering.

## Author contributions

DB, MAC, AM, SS, VS, and ADP contributed to conceptualization; VS, ADP, PS, DB, and C. Garlanda contributed to methodology; ML and DB contributed to software; VS, HON, MP, and IB performed validation and formal analysis; VS, HON, FS, TS, ML, PS, LT, IB, MP, NT, C. Gaudenzi, and C. Garlanda conducted the investigation; VS, HON, FS, ML, and IB curated data; DB, VS, and HON wrote the original draft; AM, SS, MAC, PS, ADP, and DB reviewed and edited the manuscript; HON and IB contributed to visualization; DB, MAC, and SS supervised the study; SS, MAC, DB, and PS acquired funding. All authors have read and agreed to the published version of the manuscript.

## Supplementary Material

Supplemental data

## Version 1 08/06/2021

In-Press Preview

## Version 2 09/22/2021

Electronic publication
