# Supplementary material for: SARS-CoV-2–associated ssRNAs activate inflammation and immunity via TLR7/8
Source: JCI Insight. 2021 Sep 22;6(18):e150542. doi: 10.1172/jci.insight.150542 (PMC8492321; doi:10.1172/jci.insight.150542)
Supplement: Supplemental data [file jciinsight-6-150542-s083.pdf]

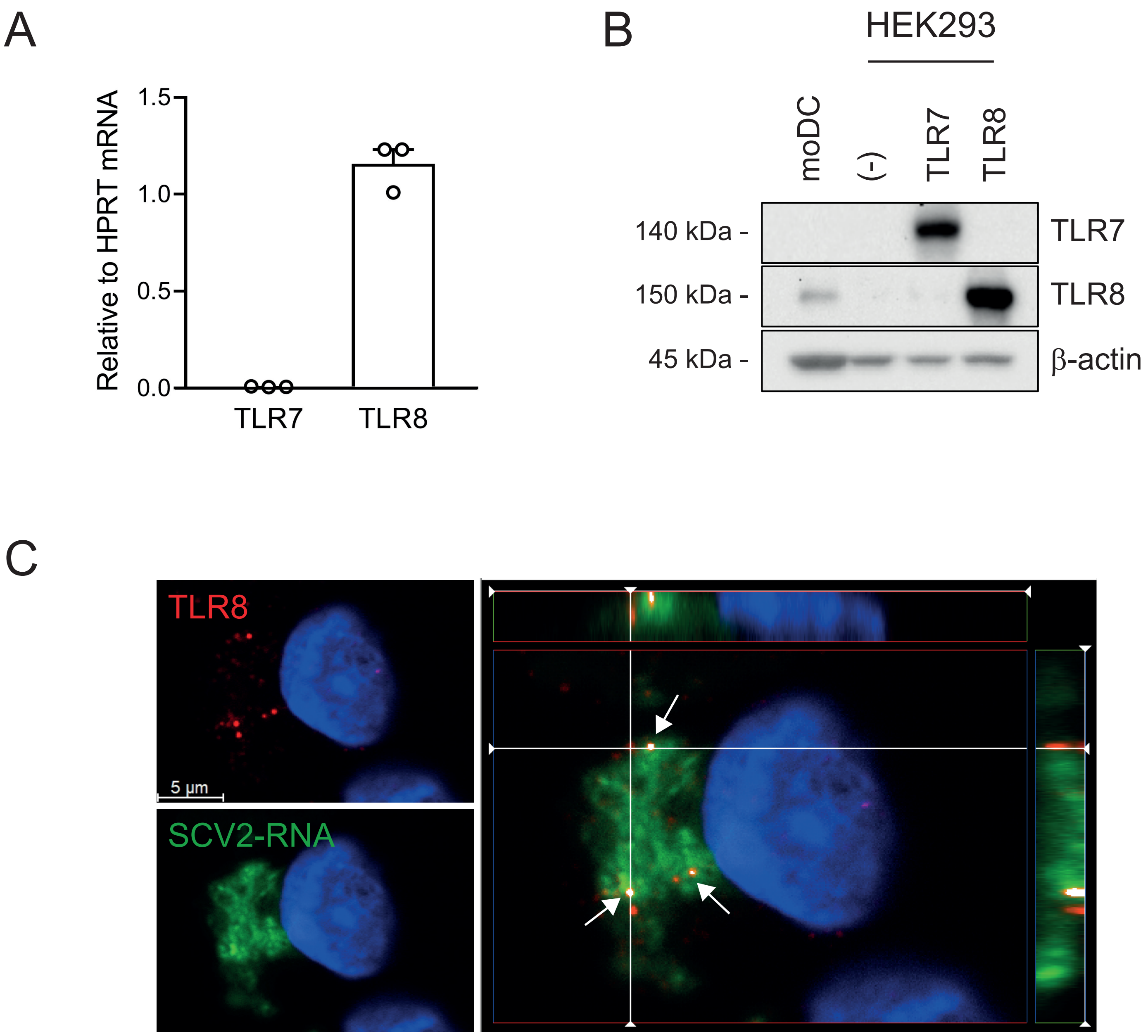

Supplemental Figure 1. TLR8 expression and SAMP colocalization in moDCs. (A) mRNA from moDCs at day 6 of culture was extracted to analyse the expression of TLR7 and TLR8 by qPCR. Data are expressed as mean ± SEM (n=3) of $2^{-\Delta Ct}$ relative to HPRT. (B) moDCs and HEK293 cells stably transfected with human TLR7, TLR8 or luciferase alone (-) were lysed and the expression of TLR7, TLR8 and β-actin was determined by Western blot. One representative fluorogram out of three is shown. (C) moDCs were stimulated with SCV2-RNA-Atto 488 for 15 minutes. After fixation and permeabilization, moDCs were stained with anti-TLR8 mAb (red) and DAPI (blue). Image on the right shows the orthogonal z reconstruction elaborated with AxioVision 3D. White dots correspond to TLR8 colocalizing with SCV2-RNA. This representative image depicts results obtained in one donor out of three; scale bar: 5 µm.

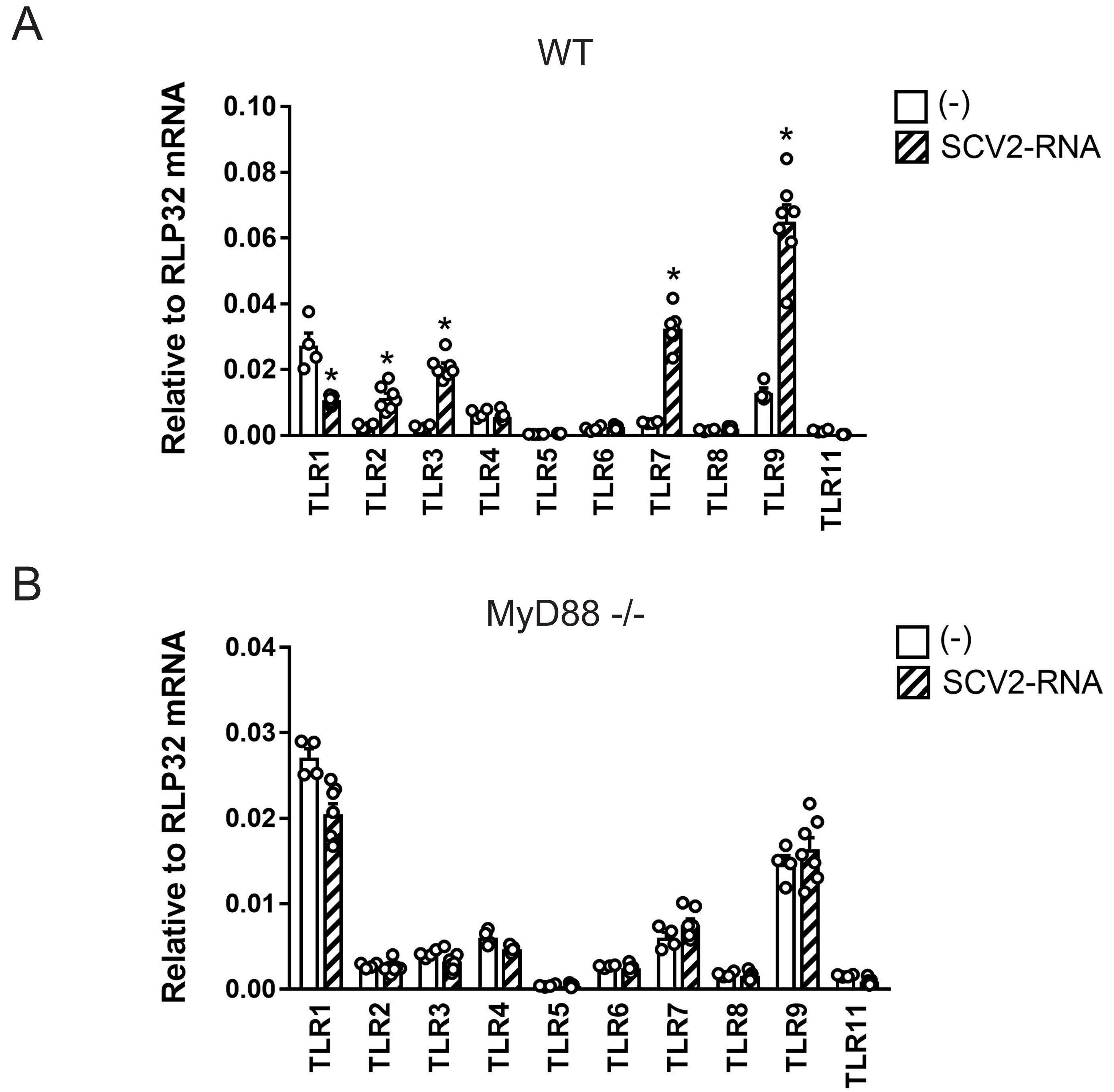

Supplemental Figure 2. SCV2-RNA stimulation affects TLR expression in WT mice. Expression of TLR mRNAs in splenocytes from WT (upper panel) or MyD88$^{-/-}$ mice (lower panel) stimulated with SCV2-RNA for 6 hours. Data are expressed as mean $\pm$ SEM ((-) n=4, SCV2-RNA n=7) of $2^{-\Delta Ct}$ relative to RPL32 of one representative experiment out of three. *P< 0.05 versus respective "(-)" by paired Student's *t* test.

Supplemental Table 1

Sequence number	Sequence	Start	Stop
1	TTTAAAATC**TGTGT**GGCTG	76	94
2	TAAAATC**TGTGT**GGCTGTC	78	96
3	C**TGTGT**GGCTGTCACTCGG	84	102
4	TTGCCTTTGGAGGC**TGTGT**	1476	1494
5	TGCCTTTGGAGGC**TGTGT**G	1477	1495
6	GCCTTTGGAGGC**TGTGTGT**	1478	1496
7	CCTTTGGAGGC**TGTGTGT**T	1479	1497
8	CTTTGGAGGC**TGTGTGT**TC	1480	1498
9	TTTGGAGGC**TGTGTGT**TCT	1481	1499
10	TGGAGGC**TGTGTGT**TCTCT	1483	1501
11	GAGGC**TGTGTGT**TCTCTTA	1485	1503
12	GGC**TGTGTGT**TCTCTTATG	1487	1505
13	GC**TGTGTGT**TCTCTTATGT	1488	1506
14	C**TGTGTGT**TCTCTTATGTT	1489	1507
15	**TGTGTGT**TCTCTTATGTTG	1490	1508
16	G**TGTGT**TCTCTTATGTTGG	1491	1509
17	**TGTGT**TCTCTTATGTTGGT	1492	1510
18	TAAATTTTTGGCTT**TGTGT**	2314	2332
19	AAATTTTTGGCTT**TGTGT**G	2315	2333
20	AATTTTTGGCTT**TGTGT**GC	2316	2334
21	ATTTTTGGCTT**TGTGT**GCT	2317	2335
22	TTTTTGGCTT**TGTGT**GCTG	2318	2336
23	TTTTGGCTT**TGTGT**GCTGA	2319	2337
24	TTGGCTT**TGTGT**GCTGACT	2321	2339
25	GGCTT**TGTGT**GCTGACTCT	2323	2341
26	TT**TGTGT**GCTGACTCTATC	2326	2344
27	**TGTGT**GCTGACTCTATCAT	2328	2346
28	ATTGTACAGAAAG**TGTGT**T	2410	2428
29	AATGAGTTCGCC**TGTGT**TG	2870	2888
30	ATGAGTTCGCC**TGTGT**TGT	2871	2889
31	TGAGTTCGCC**TGTGT**TGTG	2872	2890
32	GAGTTCGCC**TGTGT**TGTGG	2873	2891
33	GTTCGCC**TGTGT**TGTGGCA	2875	2893
34	CC**TGTGT**TGTGGCAGATGC	2880	2898
35	**TGTGT**TGTGGCAGATGCTG	2882	2900
36	AAGTGGGTGGTAGT**TGTGT**	3558	3576
37	AGTGGGTGGTAGT**TGTGT**T	3559	3577
38	GTGGGTGGTAGT**TGTGT**TT	3560	3578
39	TGGGTGGTAGT**TGTGT**TTT	3561	3579
40	GGGTGGTAGT**TGTGT**TTTA	3562	3580
41	GGTGGTAGT**TGTGT**TTTAA	3563	3581
42	GTGGTAGT**TGTGT**TTTAAG	3564	3582
43	TGGTAGT**TGTGT**TTTAAGC	3565	3583
44	GTAGT**TGTGT**TTTAAGCGG	3567	3585
45	GT**TGTGT**TTTAAGCGGACA	3570	3588
46	ATTCTTTAAGAGTT**TGTGT**	3744	3762
47	TTCTTTAAGAGTT**TGTGT**A	3745	3763
48	CTTTAAGAGTT**TGTGT**AGA	3747	3765
49	TTAAGAGTT**TGTGT**AGATA	3749	3767
50	AAGAGTT**TGTGT**AGATACT	3751	3769
51	GAGTT**TGTGT**AGATACTGT	3753	3771

52	AGTT**TGTGT**AGATACTGTT	3754	3772
53	GTT**TGTGT**AGATACTGTTC	3755	3773
54	TT**TGTGT**AGATACTGTTCG	3756	3774
55	T**TGTGT**AGATACTGTTCGC	3757	3775
56	ATTAATGCCTGTC**TGTGT**G	4426	4444
57	TAATGCCTGTC**TGTGT**GGA	4428	4446
58	ATGCCTGTC**TGTGT**GGAAA	4430	4448
59	GCCTGTC**TGTGT**GGAAACT	4432	4450
60	AGTCTTGAACGTGG**TGTGT**	5503	5521
61	TCTTGAACGTGG**TGTGT**AA	5505	5523
62	CGTGG**TGTGT**AAAACTTGT	5512	5530
63	GTGGTGTGTAAAACTTGTG	5513	5531
64	TGG**TGTGT**AAAACTTGTGG	5514	5532
65	GG**TGTGT**AAAACTTGTGGA	5515	5533
66	G**TGTGT**AAAACTTGTGGAC	5516	5534
67	TTTATTGCTACAAT**TGTGT**	6787	6805
68	TTATTGCTACAAT**TGTGT**A	6788	6806
69	ATCAACTTGTATGA**TGTGT**	7483	7501
70	CAACTTGTATGA**TGTGT**TA	7485	7503
71	AATTGGAAT**TGTGT**TAATT	7610	7628
72	TTGGAAT**TGTGT**TAATTGT	7612	7630
73	GGAAT**TGTGT**TAATTGTGA	7614	7632
74	GAAT**TGTGT**TAATTGTGAT	7615	7633
75	AAT**TGTGT**TAATTGTGATA	7616	7634
76	AT**TGTGT**TAATTGTGATAC	7617	7635
77	**TGTGT**TAATTGTGATACAT	7619	7637
78	TTAAAGTTACACT**TGTGT**T	8586	8604
79	TTACACT**TGTGT**TCCTTTT	8592	8610
80	ACACT**TGTGT**TCCTTTTTG	8594	8612
81	CACT**TGTGT**TCCTTTTTGT	8595	8613
82	ACT**TGTGT**TCCTTTTTGTT	8596	8614
83	CT**TGTGT**TCCTTTTTGTTG	8597	8615
84	T**TGTGT**TCCTTTTTGTTGC	8598	8616
85	**TGTGT**TCCTTTTTGTTGCT	8599	8617
86	ACATCAGCT**TGTGT**TTTGG	8993	9011
87	ATCAGCT**TGTGT**TTTGGCT	8995	9013
88	CAGCT**TGTGT**TTTGGCTGC	8997	9015
89	AGCT**TGTGT**TTTGGCTGCT	8998	9016
90	GCT**TGTGT**TTTGGCTGCTG	8999	9017
91	CT**TGTGT**TTTGGCTGCTGA	9000	9018
92	T**TGTGT**TTTGGCTGCTGAA	9001	9019
93	**TGTGT**TTTGGCTGCTGAAT	9002	9020
94	AGAAGCTGGTGTT**TGTGT**A	9241	9259
95	AAGCTGGTGTT**TGTGT**ATC	9243	9261
96	GCTGGTGTT**TGTGT**ATCTA	9245	9263
97	TGGTGTT**TGTGT**ATCTACT	9247	9265
98	GTGTT**TGTGT**ATCTACTAG	9249	9267
99	GTT**TGTGT**ATCTACTAGTG	9251	9269
100	TT**TGTGT**ATCTACTAGTGG	9252	9270
101	T**TGTGT**ATCTACTAGTGGT	9253	9271
102	**TGTGT**ATCTACTAGTGGTA	9254	9272
103	TAGATTATGAC**TGTGT**CTC	10509	10527
104	GATTATGAC**TGTGT**CTCTT	10511	10529

105	TTATGAC**TGTGT**CTCTTTT	10513	10531
106	ATGAC**TGTGT**CTCTTTTTG	10515	10533
107	TGAC**TGTGT**CTCTTTTTGT	10516	10534
108	GAC**TGTGT**CTCTTTTTGTT	10517	10535
109	AC**TGTGT**CTCTTTTTGTTA	10518	10536
110	C**TGTGT**CTCTTTTTGTTAC	10519	10537
111	**TGTGT**CTCTTTTTGTTACA	10520	10538
112	TGCCGTTTTAGATA**TGTGT**	10831	10849
113	GCCGTTTTAGATA**TGTGT**G	10832	10850
114	CCGTTTTAGATA**TGTGT**GC	10833	10851
115	CGTTTTAGATA**TGTGT**GCT	10834	10852
116	GTTTTAGATA**TGTGT**GCTT	10835	10853
117	TTTTAGATA**TGTGT**GCTTC	10836	10854
118	TTAGATA**TGTGT**GCTTCAT	10838	10856
119	AGATA**TGTGT**GCTTCATTA	10840	10858
120	AC**TGTGT**TATGTATGCATC	11307	11325
121	**TGTGT**TATGTATGCATCAG	11309	11327
122	AGAAC**TGTGT**ATGATGATG	11357	11375
123	AAC**TGTGT**ATGATGATGGT	11359	11377
124	AC**TGTGT**ATGATGATGGTG	11360	11378
125	C**TGTGT**ATGATGATGGTGC	11361	11379
126	**TGTGT**ATGATGATGGTGCT	11362	11380
127	AGGTATTGTTTTTA**TGTGT**	11533	11551
128	GGTATTGTTTTTA**TGTGT**G	11534	11552
129	GTATTGTTTTTA**TGTGTGT**	11535	11553
130	TATTGTTTTTA**TGTGTGT**T	11536	11554
131	ATTGTTTTTA**TGTGTGT**TG	11537	11555
132	TTGTTTTTA**TGTGTGT**TGA	11538	11556
133	TGTTTTTA**TGTGTGT**TGAG	11539	11557
134	GTTTTTA**TGTGTGT**TGAGT	11540	11558
135	TTTTA**TGTGTGT**TGAGTAT	11542	11560
136	TTTA**TGTGTGT**TGAGTATT	11543	11561
137	TTA**TGTGTGT**TGAGTATTG	11544	11562
138	TA**TGTGTGT**TGAGTATTGC	11545	11563
139	**TGTGTGT**TGAGTATTGCCC	11547	11565
140	**TGTGT**TGAGTATTGCCCTA	11549	11567
141	ATTGTGGGCTCAA**TGTGT**C	11923	11941
142	TTGTGGGCTCAA**TGTGT**CC	11924	11942
143	TGTGGGCTCAA**TGTGT**CCA	11925	11943
144	TGGGCTCAA**TGTGT**CCAGT	11927	11945
145	TGCAAGAGATGGT**TGTGT**T	12418	12436
146	AGATGGT**TGTGT**TCCCTTG	12424	12442
147	ATGGT**TGTGT**TCCCTTGAA	12426	12444
148	GT**TGTGT**TCCCTTGAACAT	12429	12447
149	CTAAT**TGTGT**TAAGATGTT	13140	13158
150	TAAT**TGTGT**TAAGATGTTG	13141	13159
151	AAT**TGTGT**TAAGATGTTGT	13142	13160
152	AT**TGTGT**TAAGATGTTGTG	13143	13161
153	T**TGTGT**TAAGATGT**TGTGT**	13144	13162
154	**TGTGT**TAAGATGT**TGTGT**A	13145	13163
155	GTGTTAAGATGT**TGTGT**AC	13146	13163
156	AAT**TGTGT**TAACTGTTTGG	14329	14347
157	AT**TGTGT**TAACTGTTTGGA	14330	14348

158	T**TGTGT**TAACTGTTTGGAT	14331	14349
159	**TGTGT**TAACTGTTTGGATG	14332	14350
160	TTTAAGGAATTACT**TGTGT**	14542	14560
161	GGAATTACT**TGTGT**ATGCT	14547	14565
162	AATTACT**TGTGT**ATGCTGC	14549	14567
163	TTACT**TGTGT**ATGCTGCTG	14551	14569
164	ACT**TGTGT**ATGCTGCTGAC	14553	14571
165	TTCTATGACTTTGC**TGTGT**	14695	14713
166	CTATGACTTTGC**TGTGT**CT	14697	14715
167	TGACTTTGC**TGTGT**CTAAG	14700	14718
168	ACTTTGC**TGTGT**CTAAGGG	14702	14720
169	TTTGC**TGTGT**CTAAGGGTT	14704	14722
170	TTGC**TGTGT**CTAAGGGTTT	14705	14723
171	TGC**TGTGT**CTAAGGGTTTC	14706	14724
172	GC**TGTGT**CTAAGGGTTTCT	14707	14725
173	C**TGTGT**CTAAGGGTTTCTT	14708	14726
174	**TGTGT**CTAAGGGTTTCTTT	14709	14727
175	GTGAAATGGTCA**TGTGT**GG	15431	15449
176	AAATGGTCA**TGTGT**GGCGG	15434	15452
177	ATGGTCA**TGTGT**GGCGGTT	15436	15454
178	GGTCA**TGTGT**GGCGGTTCA	15438	15456
179	TCTGACGATGCTGT**TGTGT**	15715	15733
180	CTGACGATGCTGT**TGTGT**G	15716	15734
181	TGACGATGCTGT**TGTGTGT**	15717	15735
182	GACGATGCTGT**TGTGTGT**T	15718	15736
183	ACGATGCTGT**TGTGTGT**TT	15719	15737
184	CGATGCTGT**TGTGTGT**TTC	15720	15738
185	ATGCTGT**TGTGTGT**TTCAA	15722	15740
186	GCTGT**TGTGTGT**TTCAATA	15724	15742
187	TGT**TGTGTGT**TTCAATAGC	15726	15744
188	G**TGTGT**TTCAATAGCACTT	15730	15748
189	CAGGGTGATGATTA**TGTGT**	15904	15922
190	AGGGTGATGATTA**TGTGT**A	15905	15923
191	GGGTGATGATTA**TGTGT**AC	15906	15924
192	GGTGATGATTA**TGTGT**ACC	15907	15925
193	TGATGATTA**TGTGT**ACCTT	15909	15927
194	GATTA**TGTGT**ACCTTCCTT	15913	15931
195	AGGCTGTTGGGGCT**TGTGT**	16235	16253
196	GGCTGTTGGGGCT**TGTGT**T	16236	16254
197	GCTGTTGGGGCT**TGTGT**TC	16237	16255
198	CTGTTGGGGCT**TGTGT**TCT	16238	16256
199	TGTTGGGGCT**TGTGT**TCTT	16239	16257
200	GTTGGGGCT**TGTGT**TCTTT	16240	16258
201	TTGGGGCT**TGTGT**TCTTTG	16241	16259
202	TGGGGCT**TGTGT**TCTTTGC	16242	16260
203	GGGCT**TGTGT**TCTTTGCAA	16244	16262
204	GGCT**TGTGT**TCTTTGCAAT	16245	16263
205	GCT**TGTGT**TCTTTGCAATT	16246	16264
206	CT**TGTGT**TCTTTGCAATTC	16247	16265
207	CATTAGTTTTCCAT**TGTGT**	16470	16488
208	ATTAGTTTTCCAT**TGTGT**G	16471	16489
209	TTAGTTTTCCAT**TGTGT**GC	16472	16490
210	TAGTTTTCCAT**TGTGT**GCT	16473	16491

211	GTTTTCCAT**TGTGT**GCTAA	16475	16493
212	TTTCCAT**TGTGT**GCTAATG	16477	16495
213	TCCAT**TGTGT**GCTAATGGA	16479	16497
214	**TGTGT**TGGTAGCGATAATG	16525	16543
215	TTTCAATTCAG**TGTGT**AGA	17499	17517
216	TTCAG**TGTGT**AGACTTATG	17505	17523
217	CTGAAGGTTTA**TGTGT**TGA	18143	18161
218	GAAGGTTTA**TGTGT**TGACA	18145	18163
219	AGGTTTA**TGTGT**TGACATA	18147	18165
220	TA**TGTGT**TGACATACCTGG	18152	18170
221	AGGTTCATCTAAG**TGTGT**G	20475	20493
222	GGTTCATCTAAG**TGTGTGT**	20476	20494
223	GTTCATCTAAG**TGTGTGT**G	20477	20495
224	TCATCTAAG**TGTGTGTGT**T	20479	20497
225	ATCTAAG**TGTGTGTGT**TCT	20481	20499
226	CTAAG**TGTGTGTGT**TCTGT	20483	20501
227	AAG**TGTGTGTGT**TCTGTTA	20485	20503
228	AG**TGTGTGTGT**TCTGTTAT	20486	20504
229	G**TGTGTGT**GTTCTGTTATT	20487	20505
230	**TGTGTGT**GTTCTGTTATTG	20488	20506
231	G**TGTGTGT**TCTGTTATTGA	20489	20507
232	**TGTGTGT**TCTGTTATTGAT	20490	20508
233	G**TGTGT**TCTGTTATTGATT	20491	20509
234	**TGTGT**TCTGTTATTGATTT	20492	20510
235	AGTCTCTAGTCAG**TGTGT**T	21592	21610
236	TCTCTAGTCAG**TGTGT**TAA	21594	21612
237	TCTAGTCAG**TGTGT**TAATC	21596	21614
238	TAGTCAG**TGTGT**TAATCTT	21598	21616
239	ATCTTAGGGAATT**TGTGT**T	22125	22143
240	TCTTAGGGAATT**TGTGT**TT	22126	22144
241	CTTAGGGAATT**TGTGT**TTA	22127	22145
242	TAGGGAATT**TGTGT**TTAAG	22129	22147
243	GGGAATT**TGTGT**TTAAGAA	22131	22149
244	ATT**TGTGT**TTAAGAATATT	22135	22153
245	TT**TGTGT**TTAAGAATATTG	22136	22154
246	T**TGTGT**TTAAGAATATTGA	22137	22155
247	**TGTGT**TTAAGAATATTGAT	22138	22156
248	C**TGTGT**TGCTGATTATTCT	22642	22660
249	**TGTGT**TGCTGATTATTCTG	22643	22661
250	ATGTCAGAG**TGTGT**ACTTG	24647	24665
251	GTCAGAG**TGTGT**ACTTGGA	24649	24667
252	ACATT**TGTGT**CTGGTAACT	24920	24938
253	ATT**TGTGT**CTGGTAACTGT	24922	24940
254	TT**TGTGT**CTGGTAACTGTG	24923	24941
255	T**TGTGT**CTGGTAACTGTGA	24924	24942
256	**TGTGT**CTGGTAACTGTGAT	24925	24943
257	TGGAGTAAAAGAC**TGTGT**T	25977	25995
258	GAGTAAAAGAC**TGTGT**TGT	25979	25997
259	GTAAAAGAC**TGTGT**TGTAT	25981	25999
260	**TGTGT**TGTATTACACAGTT	25990	26008
261	ACTGCGCTTCGAT**TGTGT**G	26347	26365
262	CTGCGCTTCGAT**TGTGT**GC	26348	26366
263	TGCGCTTCGAT**TGTGT**GCG	26349	26367

264	CGCTTCGAT**<u>TGTGT</u>**GCGTA	26351	26369
265	CTTCGAT**<u>TGTGT</u>**GCGTACT	26353	26371
266	TCGAT**<u>TGTGT</u>**GCGTACTGC	26355	26373
267	GAT**<u>TGTGT</u>**GCGTACTGCTG	26357	26375
268	T**<u>TGTGT</u>**GCGTACTGCTGCA	26359	26377
269	CCAAGAG**<u>TGTGT</u>**TAGAGGT	27453	27471
270	AG**<u>TGTGT</u>**AACATTAGGGAG	29687	29705
271	G**<u>TGTGT</u>**AACATTAGGGAGG	29688	29706

Supplemental Table 1

List of SARS-CoV-2 GU-rich sequences enriched in Interferon Induction Motif (IIM)

The reference SARS-CoV-2 genome (NC_045512, positive strand) was scanned for GU-rich ssRNA as explained in Material and Methods. Within the identified 491 GU-rich sequences, 271 were further selected based on their content of at least one "UGUGU" Interferon Induction Motif (IIM). Base numbering is based on the SARS-CoV-2 reference genome NC_045512.

Gene Name	Species	Primer Sequence (5'-3')
RPL32	mouse	Forward GCTGCCATCTGTTTTACGG<br>Reverse TGACTGGTGCCTGATGAACT
TLR1	mouse	Forward CTGTCTTCAGATGCTTACCTCC<br>Reverse GGATGCTACATTGAGTTCCTGC
TLR2	mouse	Forward CAGCTCACCGATGAAGAAGCTG<br>Reverse GAACTCTACCTCCGACAGTTCC
TLR3	mouse	Forward CGAACCTGACAGAACTCGATC<br>Reverse TTGGAGGTTCTCCAGTTGGAC
TLR4	mouse	Forward CACGTCCATCGGTTGATCTTGG<br>Reverse AGTCCTTCCATGATAGAGGGTTC
TLR5	mouse	Forward TGCTAACAAGCTGACCGTGCTC<br>Reverse TTCACAGTTGCAGACGAACTCG
TLR6	mouse	Forward GAGCTGAGAGTTCTGAGACTC<br>Reverse CCGATTGTGTGAGACATCCAG
TLR7	mouse	Forward TGATCCTGGCCTATCTCTGAC<br>Reverse CGTGTCCACATCGAAAACAC
TLR8	mouse	Forward TGGCATTTACACCCTCACAGAG<br>Reverse CATTCCACAAACGGTCAAGACG
TLR9	mouse	Forward CATCACCTGAGCCATCTGGAAG<br>Reverse GAGGTTGACCAGACCTTGGAAC
TLR11	mouse	Forward TCTCACCAGTTTCATTCTGCTGC<br>Reverse GAGTGTCCAGAGGTGATGAAGC
TNF-α	mouse	Forward TGCCTATGTCTCAGCCTCTTC<br>Reverse GAGGCCATTTGGGAACTTCT
IL-1β	mouse	Forward TGTAATGAAAGACGGCACACC<br>Reverse TCTTCTTTGGGTATTGCTTGG
IL-6	mouse	Forward GCTACCAAACTGGATATAATCAGGA<br>Reverse CCAGGTAGCTATGGTACTCCAGAA
IFN-α4	mouse	Forward TCAAGCCATCCTTGTGCTAA<br>Reverse GTCTTTTGATGTGAAGAGGTT
IFN-γ	mouse	Forward ATCTGGAGGAACTGGCAAAA<br>Reverse TTCAAGACTTCAAAGAGTCTGAGGTA
CCL3	mouse	Forward TGCCTGCTGCTTCTCCTACAGC<br>Reverse CCCAGGTCTCTTTGGAGTCAGC
CCL4	mouse	Forward TGTGCTCCAGGGTTCTCAGCAC<br>Reverse GGGTTAGCACAGATCTGTCTGC
CCL20	mouse	Forward AACTGGGTGAAAAGGGCTGT<br>Reverse GTCCAATTCCATCCCAAAAA
CCL22	mouse	Forward TCTTGCTGTGGCAATTCAGA<br>Reverse GAGGGTGACGGATGTAGTCC
CXCL10	mouse	Forward CGTCATTTTCTGCCTCATCCTG<br>Reverse CCGTCATCGATATGGATGCAGT
TRAIL	mouse	Forward CTCACATTACTGGGATCACTCG<br>Reverse CTCCCAGGATTCAATCTTCTGG
Granzyme B	mouse	Forward CCCAAAGACCAAACGTGCTTCC<br>Reverse AAGCACGTGGAGGTGAACCATC
HPRT	human	Forward CCAGTCAACAGGGGACATAAA<br>Reverse CACAATCAAGACATTCTTTCCAGT

TLR7	human	Forward TTAACCAATTGCTTCCGTGTC<br>Reverse GGTGCCCACACTCAATCTG
TLR8	human	Forward TGTGGTTGTTTTCTGGATTCAA<br>Reverse GCTCGCATGGCTTACATGA

Supplemental Table 2. Primer list used for qPCR.
